# Reduced Graphene Oxide/Poly(Pyrrole-*co*-Thiophene) Hybrid Composite Materials: Synthesis, Characterization, and Supercapacitive Properties

**DOI:** 10.3390/polym12051110

**Published:** 2020-05-13

**Authors:** Anwar ul Haq Ali Shah, Sami Ullah, Salma Bilal, Gul Rahman, Humaira Seema

**Affiliations:** 1 Institute of Chemical Sciences, University of Peshawar, Peshawar 25120, Pakistan; anwarulhaqalishah@uop.edu.pk (A.u.H.A.S.); samiullahkhalil90@gmail.com (S.U.); gul_rahman47@uop.edu.pk (G.R.); humaira@uop.edu.pk (H.S.); 2 National Center of Excellence in Physical Chemistry, University of Peshawar, Peshawar 25120, Pakistan; 3 TU Braunschweig Institute of Energy and Process Systems Engineering, Franz-Liszt-Straße 35, 38106 Braunschweig, Germany

**Keywords:** reduced graphene oxide (RGO), poly(pyrrole-*co*-thiophene) (COP), supercapacitor, energy density, power density

## Abstract

Reduced graphene oxide/poly(pyrrol-*co*-thiophene) (RGO/COP), prepared by facile in-situ oxidative copolymerization, is reported as a new hybrid composite material with improved supercapacitance performance as compared to the respective homopolymers and their composites with RGO. The as-prepared hybrid materials were characterized with ultraviolet–visible (UV–Vis) spectroscopy, Fourier-transform infrared (FTIR) spectroscopy, X-ray diffraction (XRD), scanning electron microscopy (SEM), and energy dispersive X-ray (EDX) analysis. The electrochemical behavior and energy storage properties of the materials were tested by cyclic voltammetry (CV), galvanostatic charge/discharge (GCD), and electrostatic impedance spectroscopy (EIS) techniques in 0.5 M H_2_SO_4_. The specific capacitance (Csp) for RGO/COP calculated from the CV curve was 467 F/g at a scan rate of 10 mV/s. While the Csp calculated from the GCD was 417 F/g at a current density of 0.81 A/g. The energy density calculated was 86.4 Wh/kg with a power density of 630 W/kg. The hybrid composite exhibits good cyclic stability with 65% capacitance retention after 1000 cycles at a scan rate of 100 mV/s. The present work brings a significance development of RGO/COP composites to the electrode materials for pseudocapacitive application.

## 1. Introduction

Currently, rapid energy consumption needs reliable and viable energy management from improved energy production and storage. The development of energy storage should meet strict requirements, i.e., processable size and weight to enhance the performance of portable electronic and wearable devices [1,2]. Electrochemical energy storage technologies have gained much attention because of their positive properties [3]. Energy storage technologies greatly reduce the waste of energy and play a key role to enable renewable energy resources development. Supercapacitors (SCs), promising energy storage devices, are reducing the gap between traditional capacitors and secondary batteries. SCs have attracted much attention in the 21st century due to their fascinating characteristics, such as high specific power, long life cycle, ultrafast charge/discharge rate, low cost, and eco-friendliness compared to secondary batteries [4,5].

On the basis of an energy storage mechanism, electrochemical supercapacitors are divided into two categories i.e., pseudocapacitors (PCs) and electrical double-layer capacitors (EDLCs). The energy storage in EDLC is from the reversible absorption of ions at the electrode/electrolyte interface. However, due to the limited surface area and nature of electrode materials, its capacitance and energy density are analogous to conventional capacitors [6,7,8]. On the other hand, PCs retain higher specific energy density, but limited life cycle and rapid loss of power density. Usually, carbon-based materials are employed as electrodes for double-layer capacitors, whereas the transition metal oxides and conducting polymers correspond to pseudocapacitors. At present, the actual challenges for the advancement of supercapacitors are to improve their specific energy density and enable their capability for high power density and long life cycle [9,10].

Carbon-based materials, such as chemical vapor deposited (CVD) graphene, graphene quantum dots, graphene oxide (GO), reduced graphene oxide (RGO), carbon nano tubes (CNTs), and carbon nanorods (CNRs) [11,12,13,14,15] are recently employed as advanced electrode materials for electrochemical energy storage technologies owing to the geometry and the unique structure of graphene [16]. Particularly, thin films graphene materials have shown devotion because of their binder-free processability, decent griping in between the current collector and electroactive substance, low pore volume [11,14], which are important characters for attaining the high volumetric capacitance [17]. In order to develop next-generation graphene-based materials, heteroatoms (e.g., N, S, O, P)-doped graphene is a positive approach to improve the performance of supercapacitors by introducing pseudocapacitance [18,19]. Although, heteroatom-doped and porous graphene are quite effective and successful materials for supercapacitors, the controlled thickness and uniform doping of graphene sheets with various ratios of different heteroatoms has not been achieved so far [20].

Besides this, graphene-based conducting polymers hybrid composites have shown significantly increased capacitive performance due to the additional capacitance because of the Faradic redox reactions. Consequently, the cyclic voltammetric curves of the polymer hybrid composites show a quasi-rectangular shape having redox peaks showing the existence of additional pseudocapacitance along with electric double-layer capacitance [21,22,23].

Graphene, due to its superb conductivity, greater surface to volume ratio, and exceptional electric double-layer capacitance is desirable for supercapacitors. Still, the performance of modern graphene-based supercapacitors is limited, due to the absence of the fast redox pseudocapacitance. To solve this problem, we have employed a simple strategy towards the production of multifunctional RGO/COP hybrid material based on pyrrole-thiophene copolymer (COP) covalently grafted to RGO. The combination of fast faradic pseudocapacitive COP and RGO lead to a novel electrode material for supercapacitors. The synthesis of (RGO/COP) hybrid material was carried out by first synthesizing poly(pyrrole-*co*-thiophene) (COP) by simple oxidative polymerization reaction between pyrrole and thiophene monomers. COPs were characterized with UV/Vis and Fourier-transform infrared spectroscopy (FTIR) spectroscopies, X-ray diffraction (XRD), and elemental analysis. The functionalization of RGO with COP was carried out by readily synthesized aqueous dispersion of RGO with pyrrole and thiophene monomers, followed by in-situ oxidative copolymerization, using FeCl_3_ and APS as oxidizing agents.

## 2. Experimental

### 2.1. Materials and Chemicals

Pyrrole (Alfa Aesar 98%) was double distilled prior to use. Thiophene was purchased from Sigma Aldrich chemie GMBH, Steinheim, Germany. Ammonium persulphate (APS) [(NH_4_)_2_S_2_O_8_ 99%] was purchased from EMD Chemicals. Ferric chloride (FeCl_3_ 99.5%), hydrogen peroxide (H_2_O_2_ 30%), graphite powder (98%), sulfuric acid (H_2_SO_4_ 98%), potassium permanganate (KMnO_4_ 99%), hydrazine hydrate (NH_2_-NH_2_ 99%), hydrochloric acid (HCl 37%), Sodium nitrate (NaNO_3_ 99%), and acetone (CH_3_COCH_3_ 99.5%), were obtained from Merck KGa 64271 (Darmstadt, Germany). Ethanol (C_2_H_5_OH) was obtained from Sigma Aldrich chemie GmbH (Steinheim, Germany). Doubled distilled water was used for preparation of all solutions.

### 2.2. Preparation of Graphene Oxide (GO) and Reduced Graphene Oxide (RGO)

Modified Hummer’s method was followed for graphene oxide (GO) preparation and its reduction [24,25]. An amount of 1.50 g of NaNO_3_ and 1.50 g graphite powder was added to 23 mL H_2_SO_4_, stirred for 30 min, and kept in ice bath. Then 3.0 g KMnO_4_ was slowly added to this solution. The mixture was stirred for about 2 h by keeping the temperature at 35 °C until a thick paste of dark brown color was formed. After this, 5 mL of 30% H_2_O_2_ was added in order to remove the unreacted KMnO_4_ in the paste. The yellow color ppt obtained was thoroughly washed with 1.0 **M** HCl and then washed with deionized H_2_O till the pH of the filtrate became neutral. The dark brown color product was kept in vacuum oven and dried at 40 °C till brown color solid graphene oxide sheets were obtained. For reduction (RGO formation), 1.0 g GO was taken in 50 mL water and thoroughly sonicated to obtain a homogeneous dispersion. Then 0.5 mL of hydrazine hydrate was added and transferred to a double neck round bottom flask and fitted with condenser. The assembly was kept in water bath and heated at 100 °C for 12 h. The black ppt (RGO) formed was washed thoroughly with deionized H_2_O and dried at 50 °C in oven.

### 2.3. Synthesis of RGO/COP Composite

An amount of 0.10 g of RGO was taken in 40 mL of HCl (0.10 **M**) solution and sonicated for 6 h to obtain a homogeneous dispersion. To this dispersion, 0.20 mL thiophene and 0.10 mL pyrrole monomers were added and stirred for 30 min. Then 0.20 g FeCl_3_ and 0.40 g APS were dissolved in 20 mL HCl (0.10 **M**). This oxidant solution was added drop wise to the RGO/monomers suspension. The whole mixture was kept on stirrer at ambient temperature for 24 h. Finally, the black ppt synthesized was washed several times with acetone and deionized H_2_O, and dried in oven at 50 °C.

### 2.4. Materials Characterization

The UV-Vis spectra of the as-prepared samples were recorded in NMP solvent with Varian Cary 50 UV-Vis spectrometer (Buckinghamshire, UK) in absorption mode in the range of 200–800 nm. IR affinity -1S spectrophotometer (Shimadzu, Japan) was used to for recording FTIR spectra. Surface morphological images were taken with JSM5910 (Joel, Japan) scanning electron microscope.

Electrochemical measurements were carried out with a Gamry Reference 600 Potentiostate/Galvanostatic (Louis Drive, Warminster, PA, USA) in a three-electrode assembly comprising of the synthesized materials coated on gold as a working electrode, saturated calomel electrode (SCE) as a reference, and gold counter electrode. Cyclic voltammograms (CVs) were recorded in 0.50 **M** H_2_SO_4_ in a potential range of −0.20–0.90 V at different scan rates. Galvanostatic charge discharge (GCD) curves were recorded at 0.35, 0.7, 1.8, and 2.8 Ag^−1^ current densities. Electrochemical impedance (EIS) measurements were done at open circuit potential with 5 mV ac perturbation in the frequency range of 0.1 Hz–100 kHz.

## 3. Result and Discussion

### 3.1. UV-Visible Spectroscopy

**Figure 1:**
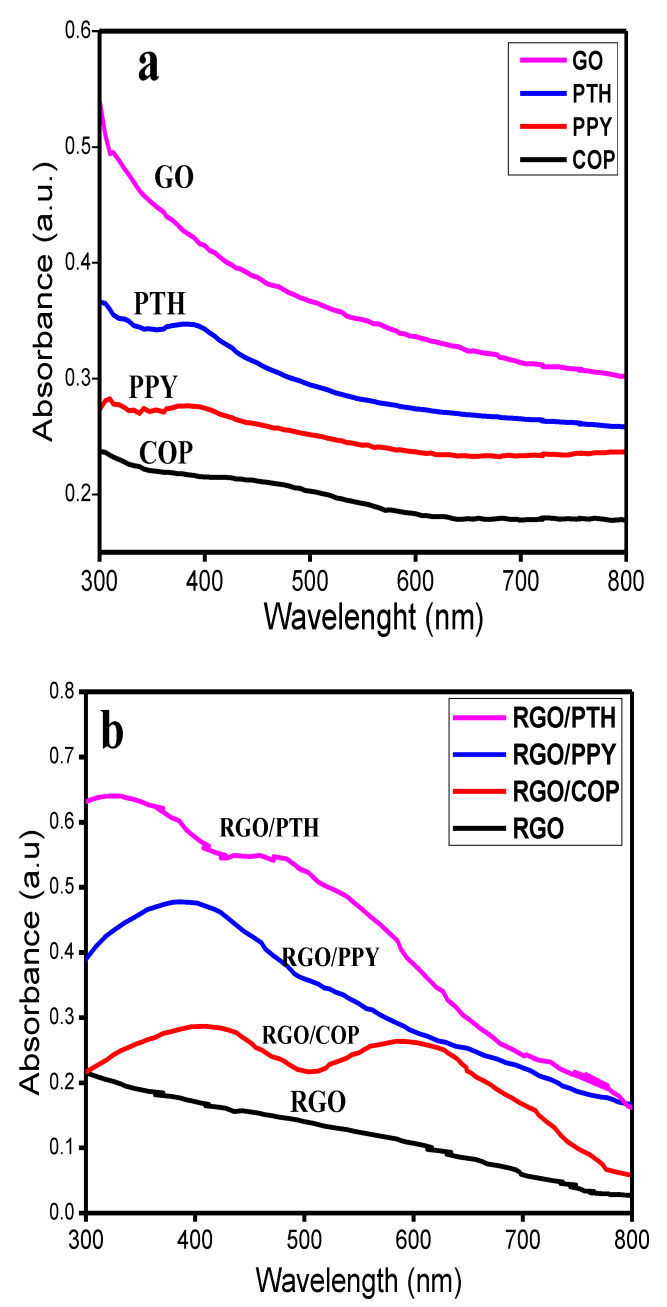
Ultraviolet/visible (UV/Vis) spectra of (**a**) graphene oxide (GO), polythiophene (PTH), polypyrrole (PPY), copolymer (COP); and (**b**) reduced graphene oxide/polythiophene (RGO/PTH), reduced graphene oxide/poly(pyrrol-*co*-thiophene) (RGO/COP), reduced graphene oxide/polypyrrole (RGO/PPY), reduced graphene oxide (RGO).

UV/visible spectra of the synthesized materials are depicted in Figure 1. The UV/Vis spectrum of GO show strong absorption, which smoothly decreases from 800 to 300 nm as a result of n→π* electronic transitions of carbonyl functional (C=O) group [26]. In the spectrum of RGO, no such absorption is observed in the UV/Vis range suggesting successful reduction of C=O functional groups of the GO [27]. The spectrum of polypyrrole (PPY) shows an absorption peak at 415 nm due to the bipolaronic state [28,29]. Whereas, RGO/PPY has a broad absorption band in the range of 300–500 nm, suggesting noncovalent interactions of the PPY with RGO sheets due to formation of RGO/PPY composite because the characteristic peak of PPY (415 nm) is shifted to a longer wavelength [27,28,29]. In the UV-Vis spectrum of polythiophene (PTH), an intense broad peak at 390 nm arising due to π–π* electronic transitions of the polythiophene chains is observed. Another absorption peak of relatively low intensity ranging from 300 to 365 nm arises due to shorter PTH chains [30]. Two intense broad peaks at 360 and 480 nm are observed in the UV-Vis spectrum of RGO/PTH. The appearance of these peaks suggests the formation of RGO/PTH composite [27,29] because the π–π* electronic transitions band of PTH (390 nm) is shifted to a longer wavelength (480 nm). The UV-Vis spectrum of the copolymer (COP) shows two absorption peaks in the range of 300–350 nm and 400–600 nm. The characteristic peaks of PPY and PTH have been shifted in UV-Vis spectrum of COP, indicating the formation of a copolymer rather than linear combination of both the polymer chains. The spectrum of RGO/COP has two broad absorption peaks ranging from 300–470 and 500–670 nm, respectively. These broad peaks may be assigned to π–π * transitions of COP chains and n→π* electronic transitions of C=O functional groups of the RGO sheets.

### 3.2. FTIR Spectroscopy

**Figure 2:**
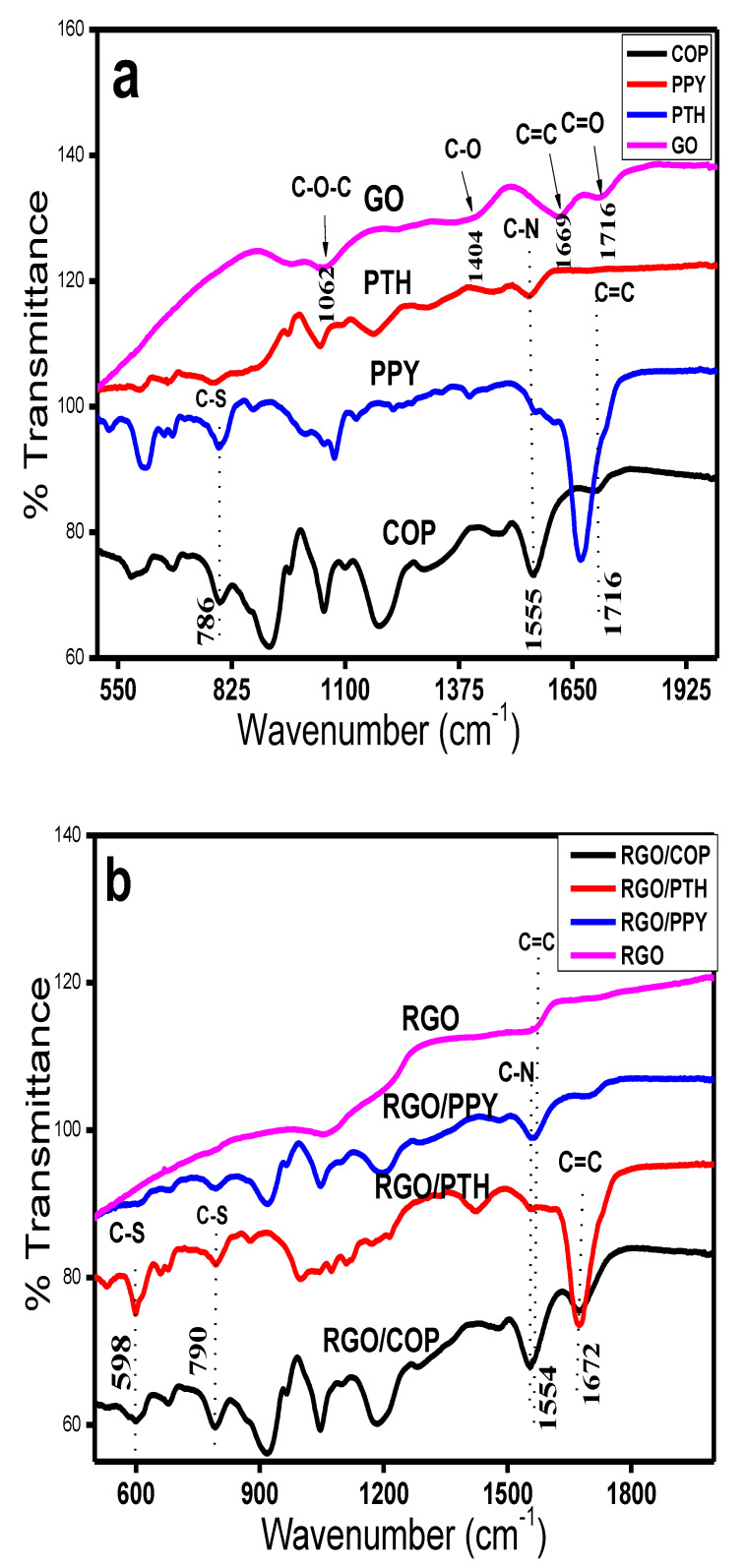
Fourier-transform infrared (FTIR) Spectra of (**a**) GO, COP, PPY, and PTH. (**b**) RGO, RGO/PPY, RGO/PTH, and RGO/COP.

Figure 2a shows the FTIR spectra of COP, PTH, PPY, and GO, while Figure 2b represents the spectra of RGO/COP, RGO/PPY, RGO/PTH composites, and RGO. Major bands along with assignments are given in Table 1. The spectrum of PPY displays typical bands at 1545 and 1455 cm^−1^ associated with stretching mode of C–N and intra/inter-ring C=C vibrations of PPY [31,32]. Whereas, a band at 1280 cm^−1^ arises due to in-plane bending vibrations of =C–H group. Another relatively intense band at 1170 cm^−1^ is associated with C–N stretching. A band at 1036 cm^−1^ corresponds to in-plane bending of C-H. Low intensity peaks at 960 and 777 cm^−1^ correspond to out-plane bending and wagging of C–H group [32]. In case of PTH peaks at 469, 697, 785, 1030, 1127, 1215, 1557, and 1665 cm^−1^ were observed. The bands at 1665 and 1557 cm^−1^ correspond to C=C symmetric/asymmetric vibrations of thiophene ring [33,34,35]. The bands at 1215 and 1127 cm^−1^ correspond to C-H in-plane and aromatic bending modes. The band at 1069 cm^−1^ refers to C–H waging mode. While the bands occurring at 790 and 609 cm^−1^ correspond to C-S out plane stretching and bending modes respectively [33,34,35]. The spectrum of COP shows bands at 1670 and 1555 cm^−1^, which correspond to conjugated C=C and C–N stretching of the PTH and PPY rings respectively. An intense band at 1455 cm^−1^ refers to the typical C=C and C–C stretching vibrations of the pyrrole ring. The band at 1280 cm^−1^ is due to in-plane bending of =C–H bond, while a peak at 1170 cm^−1^ corresponds to C–N stretching. Another band occurring at 1036 cm^−1^ may match with in-plane bending of C–H bond, while the peaks at 960 and 785 cm^−1^ match with out-of plane bending and waging modes of C–H bond [31,32,33,34,35]. The spectrum of RGO has characteristic skeletal peaks at 1560 and 1040 cm^−1^, which are assigned to C=O and C–O stretching, respectively [36,37]. The RGO/COP spectrum has bands at 1575, 1670, 1575, 1515, 1205, 1170, and 1043 cm^−1^. A broad band at 1670 cm^−1^ matches with the C=C stretching. The band at 1515 and 1575 cm^−1^ are the characteristic peaks of PPY ring given to the conjugated C–N stretching vibrations. The characteristic peak of RGO is slightly shifted to 1043 cm^−1^. Another band at 1206 cm^−1^ refers to C-C stretching. The bands at 1170 and 785 cm^−1^ belong to C-H bending and vibrations. The spectrum of RGO/PTH having peaks at 1672, 1572, 1404, 1210, 1043, 1032, 785, and 695 cm^−1^. The bands occurring at 1405 and 1670 cm^−1^ correspond to C=C bond symmetric and asymmetric vibrations of the thiophene ring [33,34,35,36,37]. While the bands at 1570 and 1040 cm^−1^ corresponding to RGO sheets are slightly shifted to 1572 and 1043 cm^−1^ in the composite material. The bands at 1215 and 1030 cm^−1^ match with C–C stretching and C-H in-plane bending modes, respectively. While the absorption peaks located at 797 cm^−1^ and 609 are assigned to C-S stretching and C-S-C ring deformation [33,34]. The spectrum of RGO/PPY shows characteristic bands at 1547 and 1454 cm^−1^, which correspond to the conjugated C–N and intra-ring and inter-ring vibrations of the C=C bond of the pyrrole ring [31,36,37]. A relatively low intensity peak at 1281 cm^−1^ is matching to =C–H in-plane deformation mode, while the band at 1172 cm^−1^ belongs to C-N stretching of the pyrrole ring. The absorption bands at 1570 cm^−1^ and 1040 cm^−1^ are due to the RGO sheets in the composite material, which confirms the formation of the RGO/PPY composites [32,37].

### 3.3. X-ray Diffraction (XRD) Study

**Figure 3:**
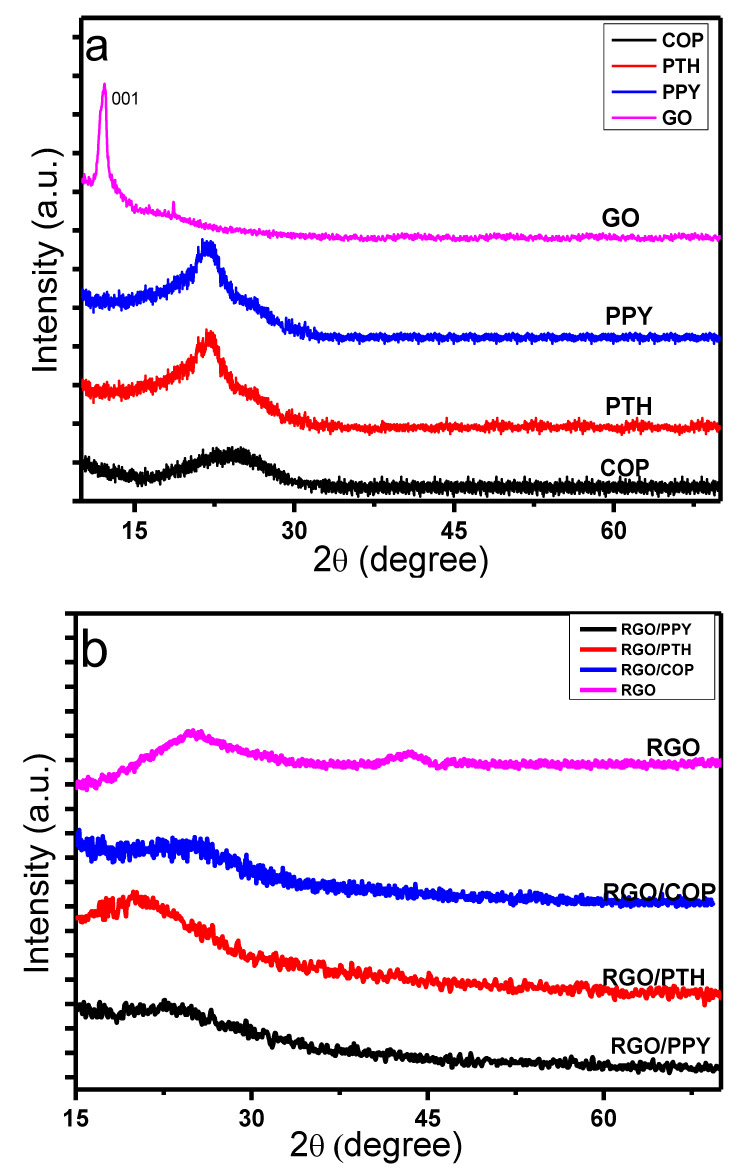
X-ray diffraction (XRD) spectra of (**a**) COP, PPY, PTH, GO; and (**b**) RGO/COP, RGO/PPY, RGO/PTH, RGO.

The XRD patterns of the synthesized materials are shown in Figure 3. The XRD pattern of GO shows a characteristic intense peak at 2θ value of 12° (JCPD no. 001) [38] indicating an inner layer d-spacing of 0.82 nm. After reduction, the typical intense peak of GO at 2θ value of 12° vanished and a broad diffraction peak ranging from 15° to 32° appeared. An additional lower intensity diffraction peak at 43° is also evident, having a d spacing of 0.4 nm (JCPD no. 002). The larger d spacing value of the GO sheets evidences the presence of water molecules among the adjacent layers of GO sheets through H-bonding, along with major functionalities, such as COOH, C=O, C–O–C, and OH group bonded covalently to sp^3^-hybridized carbon atoms, due to sp^3^ hybridization, the oxygen functionalities are arranged out of the plane of carbon backbone, consequently a more exposed structure of GO is attained. The successful reduction of GO with hydrazine (NH_2_–NH_2_) removes the oxygen functional groups from the GO intercalated films. As a result, RGO is acquired, with decreased inner layer d-spacing [38,39,40].

The XRD spectra of COP, PTH, and PPY, show typical broad peaks at 2θ values of 26°, 22°, and 23°, respectively, proposing the amorphous nature of PPY, PTH, and COP [41,42,43]. The XRD spectrum of the RGO/PPY composite displays a broad diffraction peak having 2θ value ranging from 19° to 28° [42,43], which is similar to the neat PPY diffraction peak; however, the typical diffraction peaks of RGO (15°–29°, and 43°) [39,40] disappeared in the RGO/PPY composite, suggesting the π–π interaction of the PPY with RGO sheets. In the same way the XRD patterns of RGO/PTH material displays a broad diffraction peak having 2ϴ value ranging from 17° to 28° [41], which is nearly similar to the neat PTH diffraction peak, while the typical diffraction peaks of RGO (18°–29°, and 43°) [42,43] disappeared in the RGO/PTH composite, suggesting the π–π interaction of the PTH with RGO sheets.

In the case of RGO/COP composite, a broad and relatively less intense diffraction peak of 2θ value ranging from 22° to 28° is consistent with the bear COP diffraction peak, while the typical peaks of RGO (15°–29° and 43°) vanished in the RGO/COP composite material, evidencing the π–π interactions of the COP material with the RGO films, which suggests the successful formation of a thin layer on the surface of RGO sheets during the in-situ oxidative polymerization.

### 3.4. Scanning Electron Microscopy (SEM) and Energy Dispersive X-ray (EDX) Analysis

**Figure 4:**
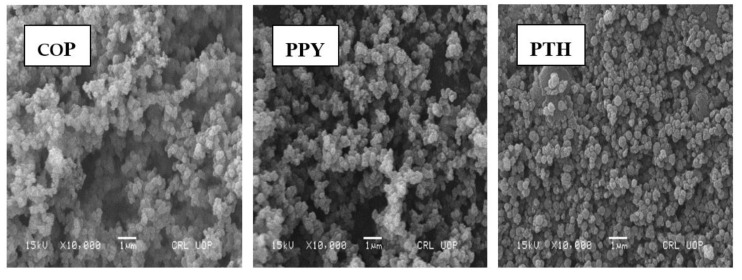

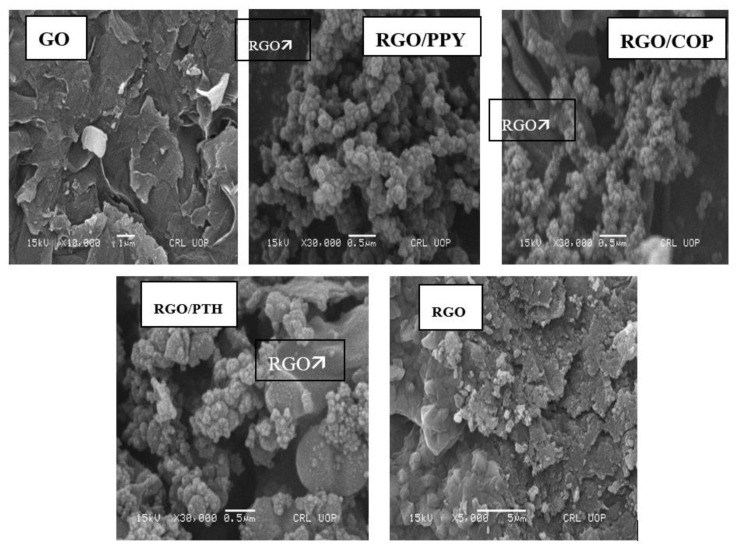
Scanning electron microscopy (SEM) images COP, PPY, PTH, RGO, RGO/PPY, RGO/PTH, and RGO/COP.

**Figure 5:**
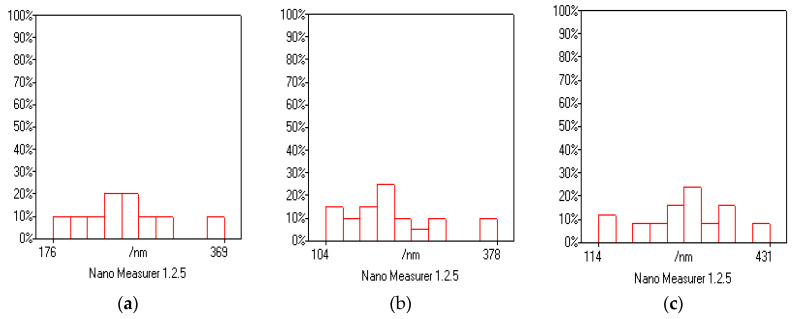
Nano Measure graphs showing particle size of (**a**) COP, (**b**) PPY, and (**c**) PTH.

Morphological and microstructural analysis was done by recording SEM images of the synthesized materials as shown in Figure 4. At high magnification, multi-layered sheet structure is observed for GO, whereas PTH displays a uniform and repeatedly granular morphology. The particle size of PTH shown in Figure 5a ranges from 104.5 to 377.9 nm. In the case of RGO/PTH composite, the repeatedly granular structure of PTH vanishes and a somewhat coarse and flaky structure is observed where PTH particles are decorating the RGO sheets, revealing the successful growth of PTH particles on the surface of the RGO. Whereas PPY has irregular granular structures, which are coagulated, the particle size of PPY shown in Figure 5b ranges from 114.5 to 365 nm. The COP morphology is cauliflower like, where the particle size shown in Figure 5c ranges from 104.5 to 350 nm. The morphology of the copolymer is coagulated and clearly different from both the PTH and PPY, suggesting the preparation of a copolymer. While for the RGO/PPY composite, the PPY particles are wrapped on the surface of RGO. For the desired composite RGO/COP, wrapping morphology is observed, where the exfoliated RGO films act as templates for COP. The COP particles cover the surface of RGO sheets. In addition, the COP have probably π–π stacking with RGO films.

The layer thickness for the COP particles on the surface of RGO sheets could be adjusted by varying the mass ratio of RGO and COP during the in-situ polymerization process.

**Figure 6:**
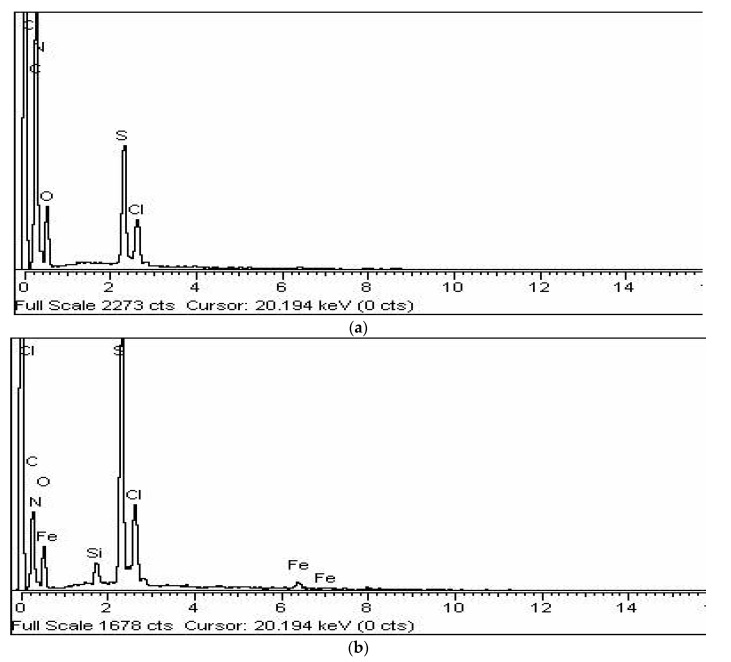
Energy dispersive X-ray (EDX) spectra of (**a**) COP (**b**) RGO/COP.

The EXD spectra describe the quantitative and qualitative aspects of the chemical constituents of materials. Figure 6a displays the EDX spectrum of the COP. The EDX spectrum shows the prsence of C, N, S, Cl, and O elements of COP. S is present in the thiohphene ring (C_4_H_4_S), while N is present in the pyrrole ring (C_4_H_5_N). The presence of oxygen is due to the oxidant APS, while Cl may come from the HCl bieng used. The carbon content arising from polypyrrole and thiophene was calculated to be 65.32%. The atomic nitrogen content was 15.83%. The atomic sulfur content was 11.27%. The presence of S and N elements in the COP adds to the successful copolymerization. Figure 6b displays the EDX spectrum of the RGO/COP. The characteristic elements of RGO, PPy, and PTH such as C, O, N, and S are observed, showing the successful RGO/COP composite material. The presence of oxygen is due to the oxidant APS, while Cl may come from the HCl bieng used. The carbon content arising from polypyrrole and thiophene was calculated to be 57.32%. The atomic nitrogen content was 10.13%. The atomic sulfur content was 15.27%. The atomic oxygen content arising from the RGO was 12.27%.

### 3.5. Electrochemical Analysis

#### 3.5.1. Cyclic Voltammetry

**Figure 7:**
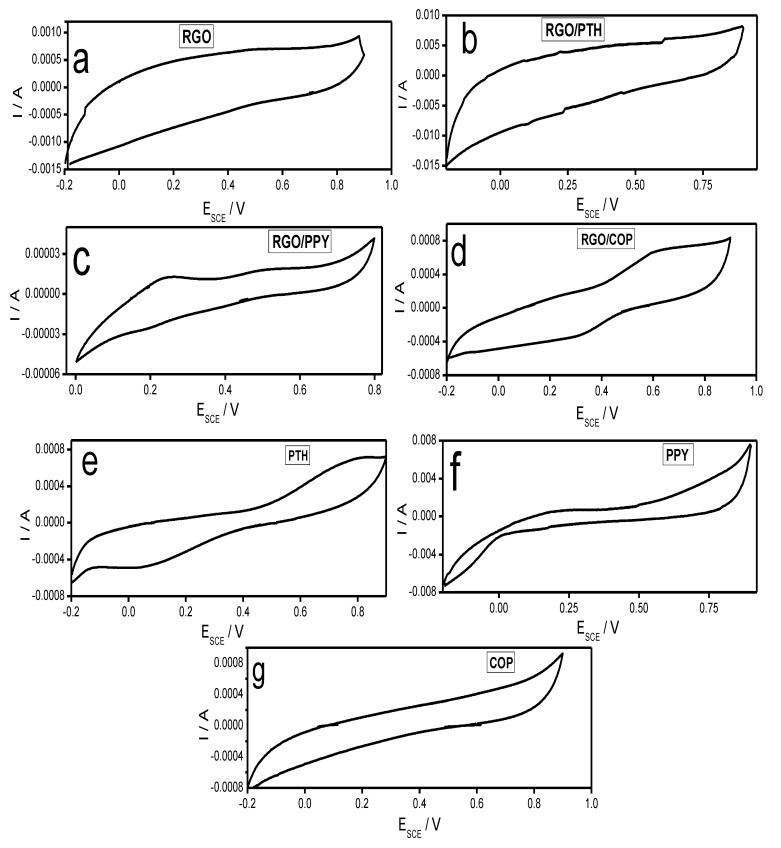
Cyclic voltammetry (CV) curves of (**a**) RGO, (**b**) RGO/PTH, (**c**) RGO/PPY, (**d**) RGO/COP, (**e**) PTH, (**f**) PPY, and (**g**) COP at 10 mV/s.

Figure 7a–g shows cyclic voltammetry (CV) curves of the synthesized materials. The CV curve of RGO in Figure 7a is nearly rectangular in shape and displays a pair of redox peaks, which is typical for the substances containing carbon with oxygen functionalities [44]. Figure 7e represents the CV curve of PTH which exhibits redox peaks due to the oxidation reduction behavior of PTH [45]. The CV curve of PTH/RGO shown in Figure 7b resembles the curve of RGO, but shows little deviation from the rectangular shape. Figure 7f displays CV curve of PPY. The shape of the curve indicates pseudocapacitive behavior of the PPY material, which is predominantly resulting from the redox behavior of PPY [46]. The CV curve of RGO/PPY in Figure 7c shows different behavior both from CV curves of RGO and PPY. The RGO/PPY curve exhibits both faradic and non-faradic behaviors offering greater area for improved capacitive than PPY [46,47]. Figure 7g demonstrates the CV of curve of COP which also exhibits irregular behavior than rectangular curve, suggesting redox behavior of the COP. Figure 7c displays CV curve of RGO/COP. The shape of the CV curve suggests pseudocapacitive behavior of the RGO/COP material, which is predominantly resulting from the redox behavior of the composite. The above-mentioned results show enhanced ion response and charge transfer performance of RGO/COP composite than that of COP and RGO [48]. The specific capacitances are evaluated from the CV curves using Equation (1) [48,49].

(1) $$C_{s}=\frac{1}{vm\;\left(Vc-Va\right)}\overset{Vc}{\underset{Va}{\int }}I_{m}dV$$

where *C_s_* denotes the specific capacitance, (*Vc* − *Va*) is potential window, *ν* is the scan rate, and *I_m_* represents the response current.

The Csp values of PTH, PPY, COP, RGO/PTH, RGO/PPY, and RGO were found to be 108, 117, 98, 79, 193 F/g, respectively, at a scan rate of 10 mVs^−1^. Whereas, RGO/COP showed the highest Csp value of 467 F/g. This might be attributed to the unique morphology and greater surface area of RGO/COP materials. 

**Figure 8:**
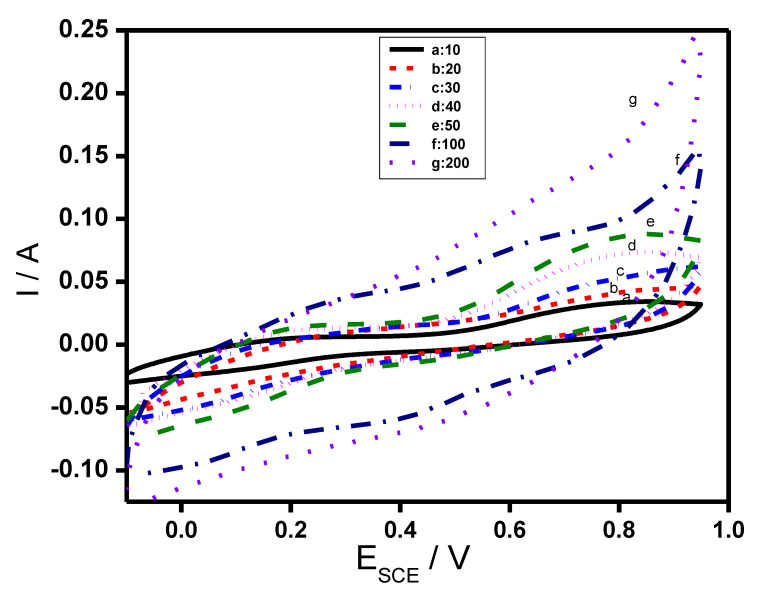
CV curves of RGO/COP composite at various scan rates as indicated.

A rectangular behavior of the CV curve is expected for an ideal double layer capacitance of the material. Deviation from the rectangular shape of the CV curve of RGO/COP, which imply the contributions of both pseudocapacitance and EDLC, where pseudocapacitance behavior is notable due to contribution from pseudo faradaic responses, a contribution from the COP materials. There is simultaneous combination of two kinds of energy storage processes. A pure electrostatic force present between the oppositely charged ions and the other is due to redox reaction occurring in the COP. A reversible redox reaction of COP was observed in the CV curve with oxidation peak at +0.610 V and reduction peak at +0.342 V in Figure 7g. The CV curve of RGO/COP enclosed larger area than those of PTh, PPy, and RGO film composites, indicating a much higher capacitance for the RGO/COP. The behavior shown by the CV curve of RGO/COP is desirable in the fabrication of capacitor electrode material. The presence of conjugated copolymer could increase the strength of 3D structure via strong π–π interaction with RGO to bear a certain extra force and is therefore responsible for the high capacitance. Besides this, there is S-doping due to thiophene ring and N-doping due to pyrrole ring in the RGO, creating synergic properties in the composite material. The composite material has a number of electroactive sites, which are responsible for the increased capacitive performance of RGO/COP [48,49,50]. Cyclic voltammograms of RGO/COP electrodes were also obtained at different scan rates ranging from 10 to 200 mV/s in the potential window of −0.20 to +0.90 V, as shown in Figure 8. All curves show distinct redox peaks demonstrating faradic behavior of the synthesized hybrid composite. The oxidation and reduction peak currents increase with the increase in scan rate showing the rapid rates of ionic and electronic transfer. In addition, with the increase in scan rate, the cathodic and anodic peaks are shifted toward lower and higher potentials, respectively. This shifting may be due to the internal resistance of the electrode. It is evident from Equation (1) that capacitance decreases with the increase in potential scan rate. At higher scan rates, the transport of ions and their accessibility to enter into the pores of electrode material, is restricted due to their hindered diffusion. Therefore, charge is stored only on the outer surface of the electrode material [51].

The specific capacitance of RGO/COP is compared with literature data for some of the polypyrrole/graphene-based and polythiophene/graphene-based composites and are summarized in Table 1. The results obtained in the present study are comparable with that reported for similar nanocomposites for supercapacitor applications. From the given literature data, it is clearly evident that RGO/COP shows much better results and is a promising candidate for supercapacitors.

**Figure 9:**
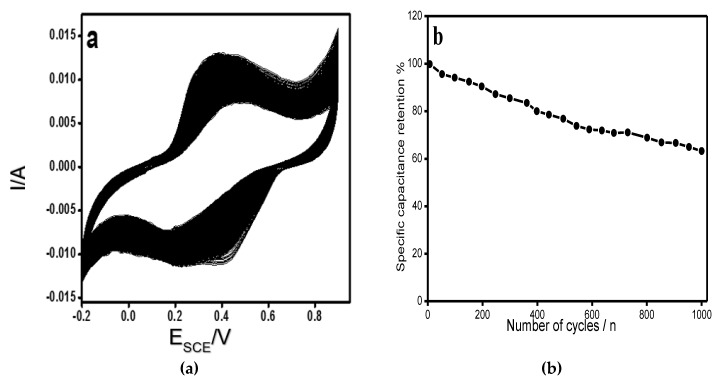
(**a**) CVs of RGO/COP at a scan rate of 100 mAs^−1^ (1000 cycles). (**b**) specific capacitance vs. cycle numbers.

For the electroactive materials, the electrochemical stability is of primary importance for their use in supercapacitors and batteries. The stability of RGO/COP composite was tested by cyclic voltammetry method for 1000 cycles and the results are shown in Figure 9. The RGO/COP retains 65% capacitance even after 1000 consecutive cycles at a scan rate of 100 mV/s.

#### 3.5.2. Galvanostatic Charge/Discharge Analysis

Galvanostatic charge discharge (GCD) behavior was studied to evaluate various capacitance parameters like specific power, specific energy, and specific capacitance of the composite material at different current densities. GCD investigation is considered more reliable for capacitance calculations than CV measurements [66]. The specific capacitance was calculated from Equation (2) [47,48,67,68,69].

(2) $$\mathrm{Csp}=\frac{I\Delta t}{m\Delta V}$$

where, I represents the current in amperes (A), m represents the mass of the electrode material in grams (g), and ΔV is the supplied voltage in volts (V).

**Figure 10:**
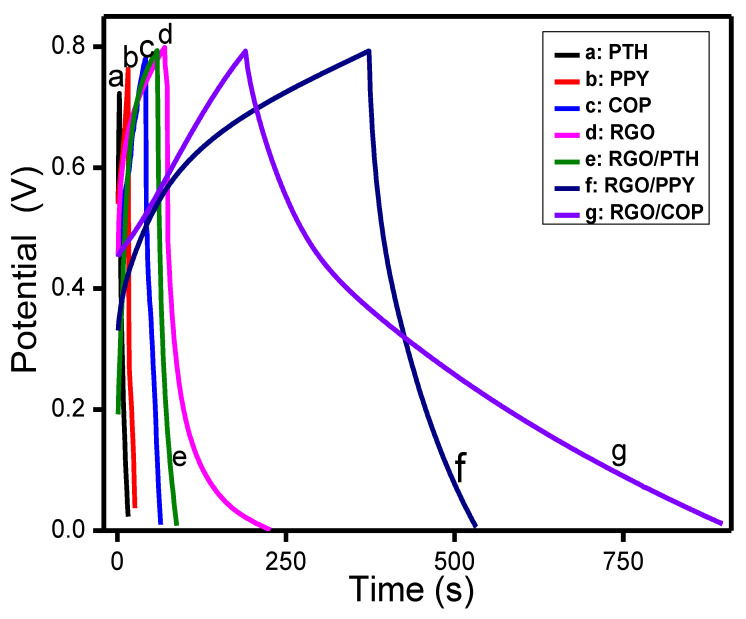
Galvanostatic charge discharge (GCD) curves of (**a**) PTH, (**b**) PPY, (**c**) COP, (**d**) RGO, (**e**) RGO/PTH, (**f**) RGO/PPY, and (**g**) RGO/COP at a current density of 0.81 A/g.

Figure 10 shows the characteristic GDC plots of PTH, PPY, COP, RGO/PTH, RGO/PPY, and RGO/COP composite coated electrode materials. The discharge time for the PTH, PPY, COP, RGO, and RGO/PTH was very similar, resulting in a comparable capacitive performance. The RGO/PPY electrode shows longer charge/discharge times. However, the RGO/COP electrode shows comparably longer charge/discharge times at a current density of 0.81 A/g, leading to approximately 60% higher charge/discharge times than all the above-mentioned electrode composites. Somewhat symmetrical charge/discharge plots are observed for the potential response of the composite. The GCD curves of the RGO/COP composite material are non-linear in nature, showing the pseudocapacitive performance of the material. The specific capacitances of PTH, PPY, COP, RGO/PTH, RGO/PPY, and RGO calculated from the charge discharge curve in Figure 10 are 48, 77, 68, 60, 163 F/g, respectively. Whereas, RGO/COP showed a Csp value of 417 F/g. These results demonstrate that RGO/COP composite exhibits higher capacitance than the aforementioned homopolymers (PPY and PTH) and their composites with RGO (RGO/PPY and RGO/PTH). The high specific capacitance obtained for the RGO/COP composite can be credited due to the fast insertion/extraction of doping ions in the RGO/COP framework, high conductivity of RGO/COP film, and larger electroactive surface area of RGO/COP material. The presence of RGO/COP composite on the electrode based on two-dimensional sheets of sp^2^-hybridized RGO permits easy contact of ions from the electrolyte. The high Csp value shows that the synthesized RGO/COP composite material is an encouraging material for application in supercapacitors [48,49,50].

**Figure 11:**
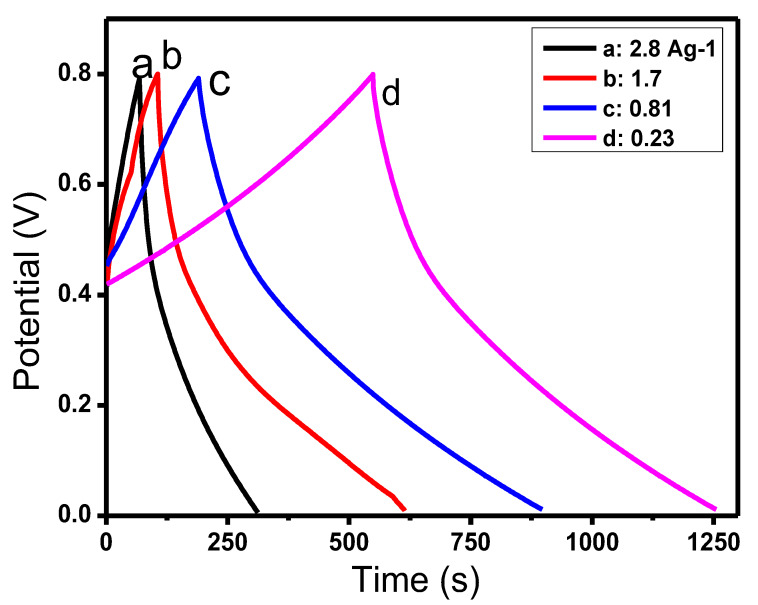
GCD curves of RGO/COP at different current densities as indicated.

The GCD measurements of RGO/COP were also carried out at different current densities ranging from 0.23 to 2.8 A/g as shown in Figure 11. The specific capacitances were observed to decrease with the increase of current densities as already reported in the literature for numerous electroactive materials.

The essential parameters for the evaluation of supercapacitance are the specific energy and specific power. The specific energy and power density are calculated using Equations (3) and (4).

(3) $$E=\frac{CV^{2}}{2M}$$

(4) $$P=\frac{V^{2}}{4MR_{S}}$$

where *C*, *V*, *M*, and *R_S_* represents the specific capacitance, voltage drop, mass of the electroactive material, and equivalent series resistance, respectively [67]. The specific energy density, calculated at current density of 0.81 A/g for the RGO/COP is 86.3 Wh/kg at a specific power of 630 W/kg.

#### 3.5.3. Electrostatic Impedance Spectroscopy

**Figure 12:**
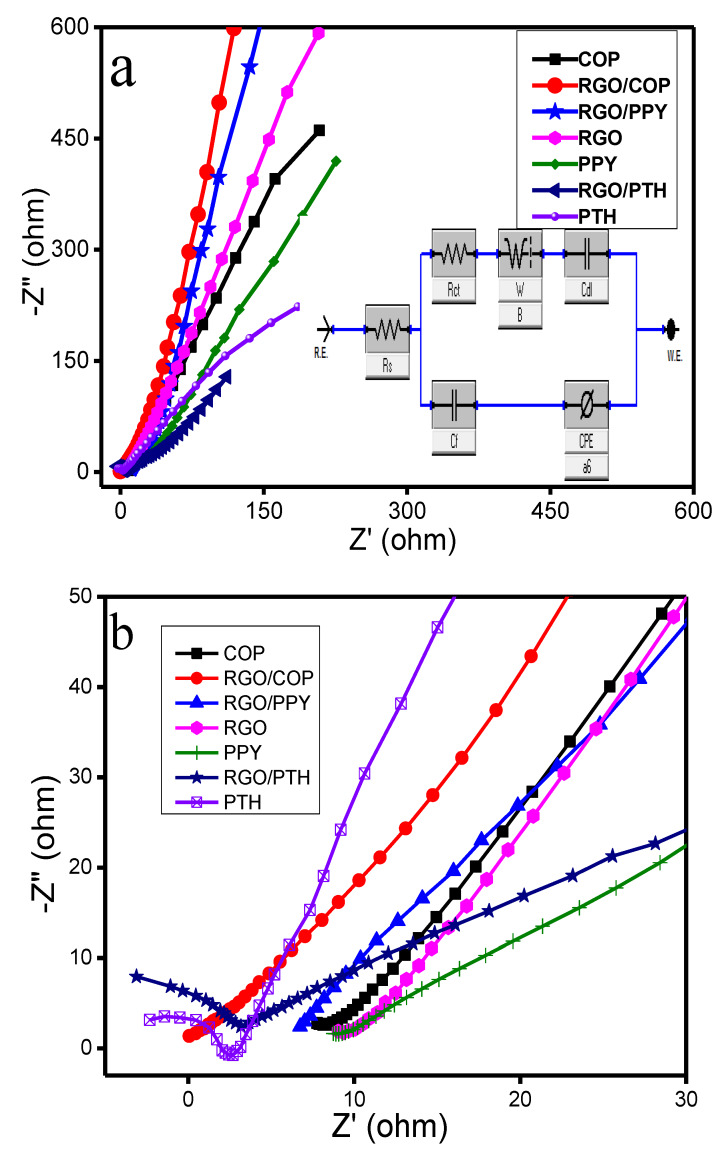
(**a**) Nyquist plots of the synthesized materials recorded in the frequency range of 0.1–10^5^ Hz with 5 mV amplitude in 0.5 **M** H_2_SO_4_ (equivalent circuit is shown in the inset) (**b**) magnification of high frequency region of plots shown in (**a**).

Electrochemical impedance spectroscopy (EIS) was carried out to verify the electronic/ionic influences and diffusion responses of the electrode material. Nyquist plots of the synthesized materials are shown in Figure 12. The Nyquist plots indicate a good supercapacitive behavior for the RGO/COP hybrid material, having a straight line with higher slope in the low frequency region. The equivalent series resistance (ESR) is determined by the intercept at the beginning of the Z′ real axis [69]. ESR includes the intrinsic resistance, ionic resistance of the electrolyte, and contact resistance at the interface of the current collector and electroactive material. For the PTH and RGO/PTH materials, semicircles were observed in the high frequency regions as shown in Figure 12b. which correspond to the charge transfer resistance (Rct) caused by Faradaic reactions and electric double layer charging at the electrode surface [70,71]. However, no semicircles were observed for the PPY, RGO/PPY, COP, RGO, and the RGO/COP hybrid material as shown in Figure 12b. From the diameter of the semicircle, Rct can be calculated [71,72]. The line at the lower frequency region is a result of ion diffusion and mass transport from the electrolyte to the electrode surface [72,73,74]. The trend of increasing slope in the low frequency region demonstrates the supercapacitive behavior for porous electrode materials. The slope in Figure 12a is highest for the RGO/COP hybrid material, as the straight line is closer to the -Z″ compared to the rest of the synthesized materials (PPY, PTH, COP, RGO/PPY, RGO/PTH, and RGO). The impedance data was evaluated by in-built electrochemical workstation (CH Inc. Model 920D) software, where the equivalent circuit is shown in Figure 12a inset. The equivalent circuit model fitting helped in determining the circuit component parameters comprising charge transfer resistance (Rct), double layer capacitance (Cdl), low frequency capacitance (Clf), solution resistance (Rs), and Warburg impedance (ZW), as summarized in Table 2. The data show improvement in the electronic and ionic conductivity of COP integrated with RGO sheets.

From the extracted parameters in Table 2, it is evident that the symmetrical device based on RGO/COP has a lower ionic and electronic resistance with a value of 2.46 Ω compared to RGO, RGO/PPY, and RGO/PTH, which are 3.56, 4.02, and 3.54 Ω, respectively. This shows the better conductivity for the RGO/COP composite electrode. This might be due to the greater capability of the COP, creating a more effective electrical connection with the underlying RGO sheets in the case of RGO/COP, which leads to the lower resistivity and faster exchange of the electrolyte ions to the electrode/electrolyte interface in the case of RGO/COP composite. In addition, RGO/COP has a lower interfacial charge transfer resistance (Rct) of 4.34 Ω, which is higher in the case of PTH, RGO/PPY, and RGO/PTH, i.e., 9.9, 6.86, and 7.68 Ω, respectively, a fact that explains the superior performance of the RGO/COP composite. Moreover, the lower Rct value of RGO/COP composite may be attributed to the highly porous structure of RGO/COP. However, the Rct value of RGO/COP, compared to RGO, is higher, showing a relatively higher resistance that limits the electrolyte ion transport to some degree. The frequency power n value lies in the range of 0 < n < 1. Closer the value of n to unity indicates ideal capacitor behavior. RGO/COP has n value 0.799, which is higher as compared to COP and RGO, which are 0.40 and 0.67, respectively, therefore showing good supercapacitive behavior.

## 4. Conclusions

We have established a facile and novel approach to the manufacture of sulphur-nitrogen-doped reduced graphene oxide material, achieved by in-situ oxidative copolymerization of the thiophene and pyrrole monomers onto RGO. The synthesized composite materials were examined in terms of chemical composition, surface morphology, and tested for electrochemical behavior. The specific capacitances for the PTH, PPY, COP, RGO/PTH, RGO/PPY, and RGO calculated from the CV curves are 108, 117, 98, 79, 193 F/g, respectively, at a scan rate of 10 mV s^−1^. Whereas the specific capacitances calculated from the galvanostatic charge discharge curves for the PTH, PPY, COP, RGO/PTH, RGO/PPY, and RGO are 48, 77, 68, 60, 163 F/g, respectively. Modification of RGO with COP lead to improved specific capacitance and energy density of the electrodes for pseudosupercapacitor applications owing to the synergic effects of RGO and copolymer. A high specific capacitance of 467 F g^−1^ at a scan rate of 10 mV s^−1^, decent cyclic stability with 63.1% specific capacitance retention. The idea of combining both pseudocapacitive copolymer and electron double-layer capacitive RGO offers favorable dedications for future portable and wearable supercapacitors and energy storage supplies in various applications.

## Figures and Tables

**Table 1:** Comparison of specific capacitance of some of the PPY/RGO- and PTH/RGO-based nanocomposites with RGO/COP.

Electrode Material	Specific Capacitance	Current Density/Scan Rate	Reference
EG-RGO/PPy	240 F g^−1^	5 A g^−1^	[52]
RGO/PPy	324 F g^−1^	1.5 A g^−1^	[53]
GO/PPy	332.6 F g^−1^	0.25 A g^−1^	[54]
rGO/PPy	389.3 F g^−1^	1.0 A g^−1^	[55]
rGO/PPy	5.5 F cm^−3^	1.6 mA cm^−2^	[56]
rGO/PPy	1685 mFcm^−2^	2 mA cm^−2^	[57]
PTh/rGO	318 F g^−1^	0.5 A g^−1^	[58]
G- PEDOT	374 Fg^−1^	0.01 Ag^−1^	[59]
GO/PEDOT	52.7 Fg^−1^	10 mVs^−1^	[60]
GO/PEDOT	64.8 mFcm^−2^	10 mVs^−1^	[61]
GO-PT derivative	296 Fg^−1^	0.3 Ag^−1^	[62]
GR-P3MT	332 Fg^−1^	0.5 Ag^−1^	[63]
GR-P3MT	240 F g^−1^	10mVs^−1^	[64]
GNPs-P3MT	215.5 F g^−1^	0.5 Ag^−1^	[65]
RGO/COP	417 F g^−1^	0.81 Ag^−1^	present work

**Table 2:** The equivalent circuit fitting values of various elements of the synthesized samples in Figure 12.

Sample	Rs	Rct	Zw	CPE	Cf	n
	Ω	Ω	S*sˆ(1/2)	S*sˆa	F	
PPY	1.86 ± 0.03	2.05 ± 0.07	0.00022	0.00132	192	0.86
PTH	4.06 ± 0.57	9.9 ± 0.61	0.0003	0.083	97	0.68
COP	1.33 ± 0.03	0.597 ± 0.01	0.007	0.00013	173	0.40
RGO	3.56 ± 0.41	0.861 ± 0.02	0.0036	0.00053	139	0.67
RGO/PTH	3.54 ± 0.6	7.68 ± 0.63	0.00045	0.0015	152	0.58
RGO/PPY	4.02 ± 0.36	6.86 ± 0.51	0.00247	0.649	132.9	0.87
RGO/COP	2.46 ± 0.06	4.34 ± 0.52	0.0022	0.0009	460	0.79
